# Disease vocabulary size as a surrogate marker for physicians’ disease knowledge volume

**DOI:** 10.1371/journal.pone.0209551

**Published:** 2018-12-27

**Authors:** Hiroaki Tanaka, Kazuhiro Ueda, Satoshi Watanuki, Takashi Watari, Yasuharu Tokuda, Takashi Okumura

**Affiliations:** 1 Graduate School of Arts and Sciences, the University of Tokyo, Meguro, Tokyo, Japan; 2 Tokyo Metropolitan Tama Medical Center, Fuchu, Tokyo, Japan; 3 Shimane University Hospital, Izumo, Shimane, Japan; 4 Japan Community Health Care Organization, Minato, Tokyo, Japan; 5 Kitami Institute of Technology, Kitami, Hokkaido, Japan; 6 National Institute of Public Health, Wako, Saitama, Japan; ESIC Medical College & PGIMSR, INDIA

## Abstract

**Objective:**

Recognizing what physicians know and do not know about a particular disease is one of the keys to designing clinical decision support systems, since these systems can fulfill complementary role by recognizing this boundary. To our knowledge, however, no study has attempted to quantify how many diseases physicians actually know and thus the boundary is unclear. This study explores a method to solve this problem by investigating whether the vocabulary assessment techniques developed in the linguistics field can be applied to assess physicians’ knowledge.

**Methods:**

The test design required us to pay special attention to disease knowledge assessment. First, to avoid imposing unnecessary burdens on the physicians, we chose a self-assessment questionnaire that was straightforward to fill out. Second, to prevent overestimation, we used a “pseudo-word” approach: fictitious diseases were included in the questionnaire, and positive responses to them were penalized. Third, we used paper-based tests, rather than computer-based ones, to further prevent participants from cheating by using a search engine. Fourth, we selectively used borderline diseases, i.e., diseases that physicians might or might not know about, rather than well-known or little-known diseases, in the questionnaire.

**Results:**

We collected 102 valid answers from 109 physicians who attended the seminars we conducted. On the basis of these answers, we estimated that the average physician knew of 2008 diseases (95% confidence interval: (1939, 2071)). This preliminary estimation agrees with the guideline for the national license examination in Japan, suggesting that this vocabulary assessment was able to evaluate physicians’ knowledge. The survey included physicians with various backgrounds, but there were no significant differences between subgroups. Other implication for researches on clinical decision support and limitation of the sampling method adopted in this study are also discussed, toward more rigorous estimation in future surveys.

## 1 Introduction

The evolutionary growth of the AI technology has revived the ventures toward prophetic clinical decision support systems, in the field [1, 2]. When building such predictive systems, assessing the amount of physicians’ knowledge for rare diseases is one of the keys to making them helpful and instructive. Recognizing what physicians do and do not know would enable such a system to provide supplementary knowledge and highlight potential unconscious biases on the part of the physician. To complement clinical medicine in this way, the system would need to assess the physician’s knowledge to compensate for the limit of human knowledge.

Surprisingly, only a small number of studies has attempted to quantify the amount of disease knowledge that a physician may have. Ramsey et al. exhibited that physicians’ medical knowledge gradually declines over time [3]. Gordon found that approximately 24,000 different terms had been used in medical publications to name 3,700–3,800 specific diseases [4]. However, no study has yet attempted to answer the simplest question of all: How many diseases do physicians know about? The World Health Organization estimates that 20,000–30,000 diseases have been discovered [5]. Given this body of disease knowledge, the number of diseases that physicians actually know about is a critical question that is foundational to the development of clinical decision support systems. It is also an important question when considering the reliability of statistics related to rare diseases, such as their incidence and prevalence, because it is clearly impossible for physicians to correctly report diseases of which they are unaware.

The absence of such a fundamental statistic can be ascribed to the absence of a standard methodology for quantifying physicians’ disease knowledge. A straightforward approach to estimating this would be to simply ask physicians about each disease and analyze the answers. As it would be infeasible to ask about all possible diseases, sampling would be necessary. However, framing such questions is not as simple as it might appear. Multiple choice questions, as used in the national license examinations for physicians, might work to test their knowledge, but licensed physicians become too busy to readily answer license-examination-style questionnaires. There is a need for something simpler to answer that will make easier to recruit the test participants and hence produce better statistical result. Appropriate sampling is another challenge: there is no consensus on how to sample diseases for such a questionnaire, or on estimating the population mean.

Accordingly, this study explores a potential method for assessing physicians’ disease knowledge by evaluating the utility of the *vocabulary assessment* techniques used in linguistics for this task. Some vocabulary assessment tests have been developed as a simple way of assessing the vocabularies of foreign language learners. Hence, they have the desirable properties of being easy to answer, convenient to conduct, and cheat-proof. This is a longstanding field of research and has produced an objective approach, supported by many empirical studies that could help to assess the physicians’ disease knowledge.

This paper is organized as follows. Section 2 presents an overview of the vocabulary assessment tests and then explores a method to apply this concept to the assessment of disease knowledge, clarifying the design conducted to validate this conjecture. Section 3 presents the survey’s results with the method in the previous section, which are then analyzed in Section 4. Section 5 discusses the remaining issues, before Section 6 concludes the paper and proposes future research directions.

## 2 Materials and methods

### 2.1 Vocabulary assessment for disease knowledge estimation

Physicians need to have several different types of knowledge about each disease [6]. First and foremost, they need to know what it looks like, i.e., its clinical symptoms. Also, they need to understand how it develops, namely the disorder’s etiological background, as well as the diagnostic criteria. They must also understand its epidemiology: how frequently and at what age it is likely to occur. Knowing what treatment options are available is also essential. However, physicians may sometimes encounter patients with rare diseases that they only know by name, meaning they need to refer to the literature to have a foundation for providing medical care.

As this illustrates, physicians can have different degrees of disease knowledge. Some types of diseases are easy for physicians to recognize and manage, whereas there are others that they only know by name. When assessing disease knowledge, it would be reasonable to determine the size of the latter group first.

In this context, linguistics have discovered that there are two types of vocabulary, *productive vocabulary* that people can use and *receptive vocabulary* that they can only recognize [7]. They have also devised methods of assessing the size of both receptive and productive vocabularies [7]. The simplest approach to assessing receptive vocabulary size is to randomly sample some words from a dictionary and ask test participants if they know these words [7]. The vocabulary size can then be estimated by extrapolating the proportion of positive responses for the sampled items to the entire dictionary.

This technique was, however, developed to determine the vocabulary size of language learners and to guide their learning process. In the following sections, we investigate how this idea can be transferred to assessing physicians’ disease knowledge.

### 2.2 Validity of self-assessment tests

This basic vocabulary assessment methodology depends on the participant’s self-assessments and cannot tolerate overstating their vocabulary knowledge. Accordingly, Meara and Buxton proposed adding fictitious words to the questionnaire and penalizing positive responses to these fictitious items [8]. In the formula below, *f*_*k*_ denotes the proportion of words known, *f*_*h*_ denotes the proportion of positive responses, and *f*_*na*_ denotes the proportion of positive responses to fictitious *pseudo-words*. They demonstrated that this simple trick can correct for overstatement, giving results that are strongly correlated with the estimates of vocabulary size produced by the more rigorous multiple choice questions that have traditionally been used in the field.

$$\begin{array}{c} f_{k}=\frac{f_{h}-f_{na}}{1-f_{na}} \end{array}$$ (1)

Although multiple-choice questions can be used for knowledge assessment, it is not easy for preparing a set of test questions with consistent quality. Even with all the costs involved, we still obtain only a statistical estimate, not a direct measurement. In addition, such tests are burdensome for test participants. The pseudo-words approach has the advantage of simplicity in that it requires a limited set of pseudo-diseases. However, pseudo-diseases can be quickly identified using a search engine. Accordingly, to prevent potential cheating, computer-based tests must be avoided and paper-based assessments are preferred.

### 2.3 Question selection—Word familiarity approach

Another issue with this simplified approach to vocabulary assessment is that the estimates depend on how the sampled words are selected [9]. Accurate results require a large number of samples, but it is important to keep the questionnaire short. Amano and Kondo addressed this problem by introducing the concept of *word familiarity*. First, they built a dictionary comprising slightly less than 90,000 words and asked participants to assess how familiar they were with them on a 7-point scale, averaging the scores for each word in the dictionary among participants. The higher the familiarity score, the better the word was known. Then, by identifying a familiarity threshold for the words in the dictionary, they estimated the average vocabulary size, based only on the limited number of samples included in the questionnaire. In their study, they had 40 participants and asked them about words that had been sampled from the sorted list. They then fitted a logistic curve to the proportion of positive answers for each word using the iteratively reweighted least squares method. The average vocabulary size was then estimated from the point on the curve where the ratio was 50% (i.e., word that 50% of the population knew).

We conjecture that this vocabulary assessment approach can be applied to assessing the number of diseases that physicians know, particularly if the mental lexicon for most diseases behaves like a receptive vocabulary. The questionnaire is kept short by assuming a certain distribution for physicians’ disease knowledge, as randomized sampling would otherwise be inefficient. This assumption is reasonable because physicians’ knowledge of a particular disease is strengthened if they encounter it on a daily basis. In this way, we may use the disease prevalence and incidence as a substitute for disease familiarity. However, it is not practical to compile the statistics for all the diseases in the vocabulary, including rare diseases. In addition, some diseases are rare but are still well known among physicians because of their importance in clinical medicine. Although diseases familiarity is highly correlated with disease prevalence in the upper region of the vocabulary, disease prevalence is not a perfect dataset, particularly for rare diseases.

As a result, we propose to replace the disease familiarity data with *search engine statistics*. Note, however, that these statistics have unwanted biases, for example giving abnormally high scores to diseases that have affected a celebrity (e.g. Lou Gehrig’s disease) or have appeared in the news for some reason. To filter out the noise, we also propose counting the number of times diseases are mentioned in the medical literature, not the entire Web, by searching for the English names of diseases but specifying Japanese pages as the search target. Because websites intended for the general public will only give Japanese names for diseases, whereas academic websites are likely to list diseases in both Japanese and English for future reference, this simple heuristic approximates the disease familiarity in academia using only publicly available search engines.

The search engine statistics were obtained as follws. First, we prepared a disease names dictionary using the disease knowledge base we have been developing [10, 11]. Second, the names in the dictionary were normalized to yield better search engine hit rates by eliminating prepositions such as “with” and “of” and manually removing disease names that do not have equivalents in Japanese. Third, we utilized the *Bing* search engine to search for English disease names in Japanese web pages. Finally, the search results were analyzed to extract the estimated total number of matches (pages) for each query, and this was used as the familiarity measure for that disease. On the basis of these approximated familiarities, we planned to identify the boundary between the diseases that the average physician did and did not know.

**Fig 1:**
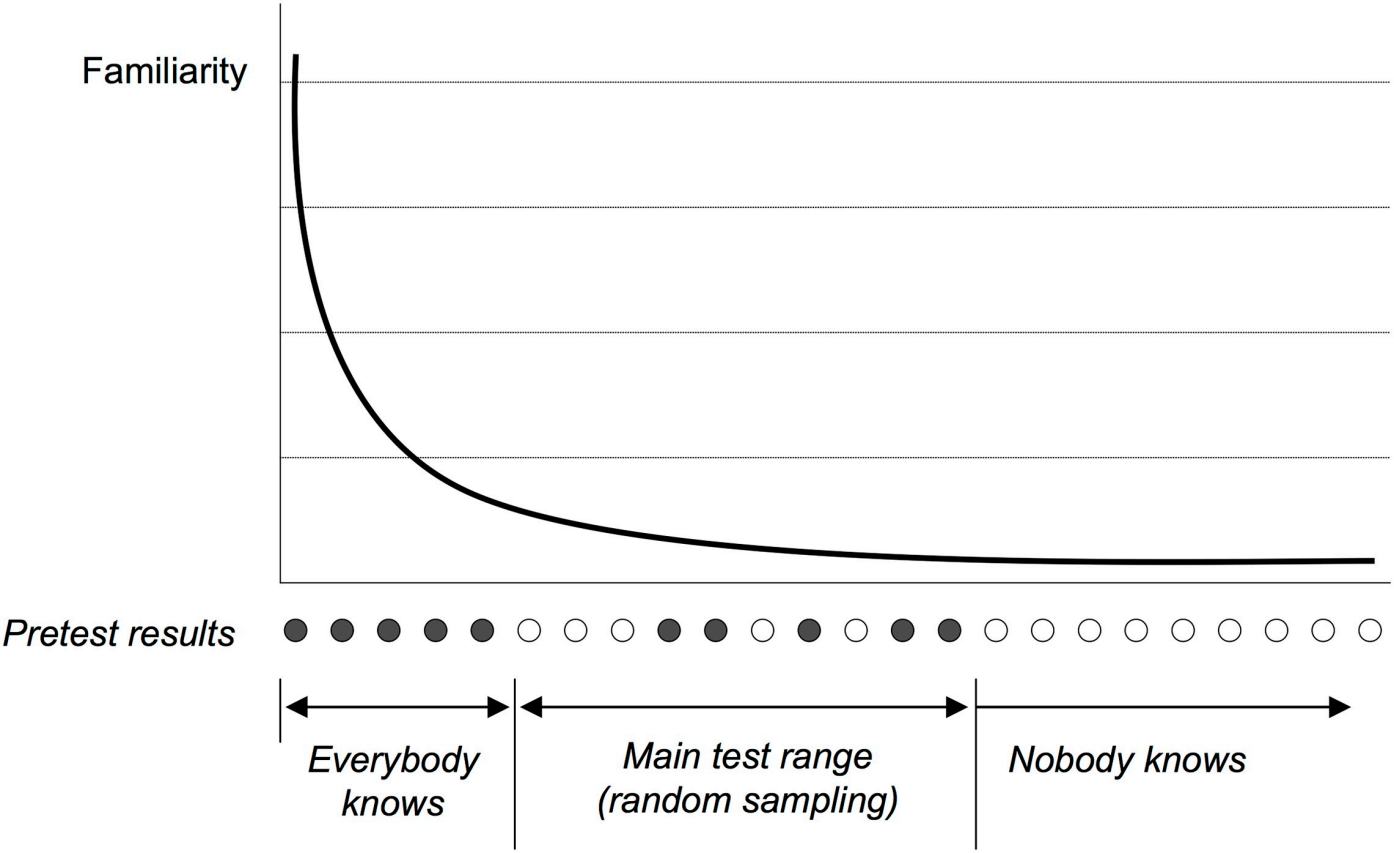
Disease familiarity distribution, showing the disease knowledge boundary.

Fig 1 illustrates the overview of this procedure. We assumed that the disease familiarity distribution was uneven, including common diseases which almost everyone knows about and rare diseases that even the physicians were unlikely to have heard of. As a result, if we could determine the extent of the *everybody knows* range (shown by black circles), those diseases could be omitted from questionnaire. Likewise, determining the *nobody knows* range (shown by white circles) would allow that range also to be skipped, allowing us to focus on the *main test* range. To this end, we performed a *pretest* to determine these boundaries where we asked physicians about diseases sampled from the entire list at regular intervals.

For the pretest, it was necessary to test the entire range, but there is a trade-off between the sampling interval and the size of the questionnaire. Using a small sampling interval would mean the pretest took a long time. However, using a large sampling interval would compromise the granularity of the range estimate. Consequently, we chose a hybrid approach: the entire range was sampled using a large interval: then, the upper part of the range was resampled at higher resolution to determine the lower bound of the *everybody knows* range with finer granularity.

Another concern was the information embedded in the disease names on the questionnaire. If a disease name was self-explanatory, the test participants might think they knew about it, even though they were seeing the name for the first time.

**Fig 2:**
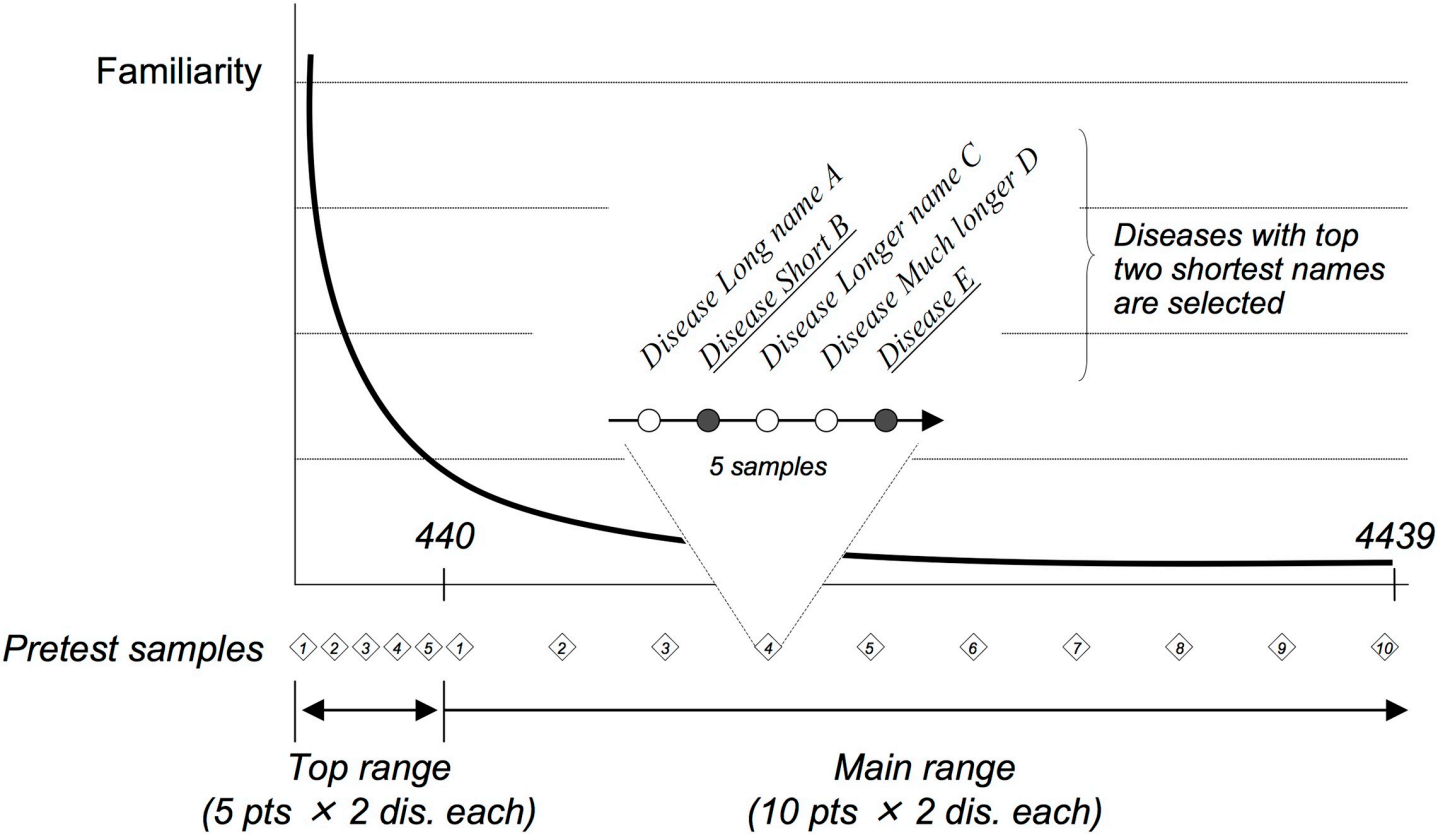
Pretest design: Breakdown of 30 samples used.

To avoid this, we used a simple heuristic to select disease names that were not self-explanatory. This involved sampling five diseases around each sampling point, including the two diseases immediately above and below the central point, and then selecting the two diseases with the shortest names among these five candidates. Using this strategy, we sampled 20 diseases as pretest questions from the complete list of 4,439 diseases, together with 10 diseases from the top 440 diseases in the list, sorted by search engine statistics. Fig 2 illustrates the design of the pretest. We then asked five physicians, including three of the authors (S.W., T.W., and T.O.), how much they knew about each of the resulting 30 diseases, on a 5-point scale (explained in the next subsection). The results were then analyzed to determine the main test range, after some manual filtering: we discarded one physician’s response as an outlier, because he claimed to know all the diseases on the list. We also discarded the responses for certain diseases that turned out to be different names of well-known diseases. Note that even after this filtering process, at least one disease out of each pair was retained, preserving the sampling interval used for the pretest.

The result of the pretest indicated that the lower bound of the *everybody knows* group was between 880 and 1,320 diseases, and the upper bound of the *nobody knows* group was between 2,650 and 3,090. We assumed that the actual bounds were in the middle of these ranges, and we set the range for the main test as between 1,100 and 2,850. In the main test, we randomly sampled 20 diseases from this range.

### 2.4 Questionnaire design

**Fig 3:**
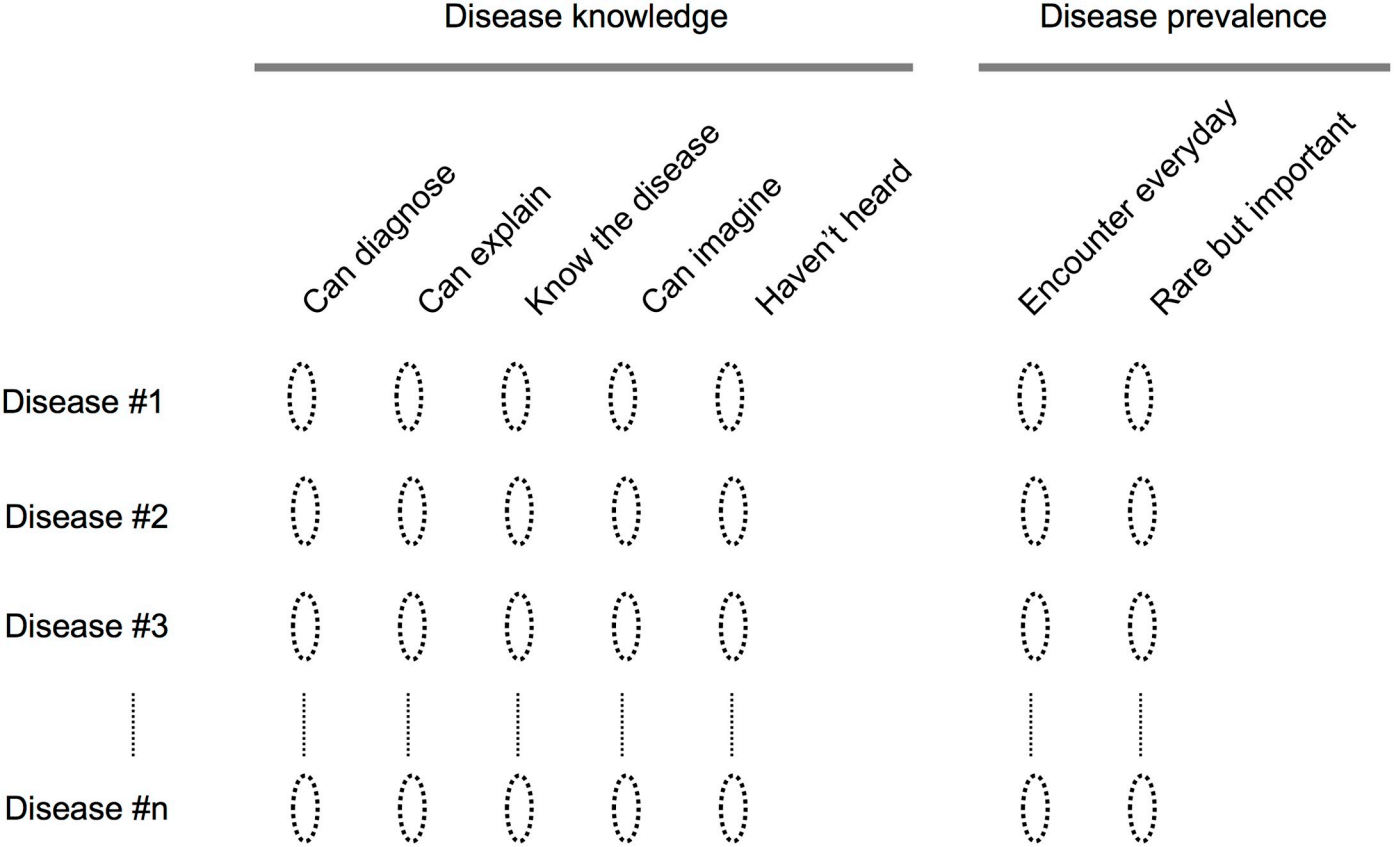
Self-assessment questionnaire.

Although the question looks simple, i.e., whether or not somebody knows about a certain disease, it can still potentially confuse a test participant because the verb *know* is not self-evident. The idea of *knowing a disease* must be defined. A simple approach to avoiding this issue is to utilize a well-defined scale, which also helps to make answering easier by minimizing the decisions needed. Another objective is to differentiate the productive and the receptive vocabulary. To deal with these issues, we devised a 5-point clinical disease knowledge scale for the questionnaire as shown in Fig 3. This scale represents the degree of disease knowledge, awarding the highest possible score if physicians can diagnose the disease. In between the *can diagnose* and *haven’t heard*, there is a spectrum of disease knowledge. Even if physicians cannot make a firm diagnosis, they might still be able to explain the disease to some extent, which would fail into the *can explain* category. Likewise, physicians might only recognize the disease by name, which would fall into the *know the disease* category. Finally, *can imagine* category may appear confusing to those without a medical background, but it represents the case where the disease name is self-explanatory, such as *primary headache*.

The selective sampling of rare diseases proposed will be necessary to precisely estimate physicians’ disease vocabularies. However, to validate their self-assessments of disease knowledge, it is reasonable to compare their responses for common diseases (with which all physicians are familiar) with their responses for rarer diseases. Consequently, along with the 20 diseases chosen as above, we also sampled another 10 diseases from the topmost region of the generated list. In addition, we invented 10 diseases with plausible names, giving us a total of 40 items to use in the questionnaire. We also asked the subjective prevalence and clinical importance of each disease for future reference.

In the preamble of the questionnaire, we requested the participants for the following personal information: (i) number of years passed since graduation, (ii) specialty, (iii) professional certification, and (iv) institution type. The whole questionnaire was designed to be processed by an optical mark reader for efficiency. An informed consent notification was also included at the beginning of the questionnaire so that only those who had agreed to participate in the study could proceed.

### 2.5 Survey settings

When conducting the actual survey, we avoided having participants return the questionnaire by mail in order to prevent potential cheating by using search engines. This was also important in protecting the questionnaire from leaking, because the pseudo-diseases were key components of the study and it was time-consuming to come up with plausible names. Instead, we called for volunteers in several seminars conducted at different types of medical institutions, to complete the survey.

Four institutions participated in the survey: Shiga Medical Center Research Institute (indicated as Shiga, hereafter), Japan Community Health Care Organization (JCHO), Shimane University Hospital Postgraduate Clinical Training Center (Shimane), and Sunagawa City Medical Center (Sunagawa).

## 3 Results

### 3.1 Response statistics and participant attributes

Table 1 summarizes the responses to the survey, conducted in four institutions. Of the 109 questionnaires distributed to the seminar attendee, 102 valid responses were received, for a response rate of 93.6%. There were striking variations in the participants across institutions. At Shiga, several attendees were with more than 20 years’ experience. JCHO had the most balanced distribution, including new graduates and senior physicians, whereas Shimane predominantly hosted new graduates. As a result, the survey covered a wide range of experience level.

**Table 1:** Summary of responses for each survey setting.

Seminar location	Attendee	Yearly Breakdown						Total Response
		1-2	3-5	6-10	11-20	> 21	NA	
Shiga Medical Center Research Institute	24	0	0	0	7	14	0	21 (87.5%)
Japan Community Health Care Organization	46	12	7	7	10	6	1	43 (93.5%)
Shimane University Hospital Postgraduate Clinical Training Center	22	18	0	0	2	2	0	22 (100.0%)
Sunagawa City Medical Center	17	15	0	0	0	1	0	16 (94.1%)
Total	109	45	7	7	19	23	1	102 (93.6%)

Tables 2–4 break down the responses in terms of the participants’ attributes. Table 2 shows the respondents’ specialties, most of which were either internists or residents, including only a limited number of surgeons. Table 3 shows the certifications held. Note that some physicians had multiple certifications, meaning that the totals for this table disagreed with those of the other tables. Hereafter, we only consider the highest certification held by each respondent. Some mistakes were made when answering this question, in that some other physicians identified themselves as *registered*, even though this only applies to anesthesiologists in Japan. These responses were manually corrected by the authors. Likewise, the institution types shown in Table 4 were corrected when the answers included evident mistakes that contradicted the context of the seminar.

**Table 2:** Participant profiles by specialty.

Category	Specialty	Shiga	JCHO	Shimane	Sunagawa	Subtotal	Total
Internal	General internal medicine	1	14	3	1	19	44
	Respiratory medicine	0	3	0	0	3	
	Cardiology	3	0	1	1	5	
	Gastroenterology	1	2	0	0	3	
	Nephrology	0	1	0	0	1	
	Neurology	1	2	0	0	3	
	Diabetology	2	1	1	0	4	
	Hematology	1	0	0	0	1	
	Infectious diseases	0	2	0	0	2	
	Rheumatology	0	1	0	0	1	
	Pediatrics	0	1	1	0	2	
Surgical	Surgery	0	1	1	0	2	4
	Orthopedic surgery	0	1	0	0	1	
	Otorhinolaryngology	0	0	0	0	0	
	Urology	0	0	0	0	0	
	Ophthalmology	0	0	0	0	0	
	Dermatology	1	0	0	0	1	
	Obstetrics and gynecology	0	0	0	0	0	
Psychiatry	Psychiatry	0	0	1	0	1	1
Other	Anesthesiology	0	0	0	0	0	15
	Radiology	0	0	0	0	0	
	Pathology	3	0	0	0	3	
	Emergency medicine	0	1	0	0	1	
	Other	8	3	0	0	11	
Resident	Resident	0	10	14	14	38	38
Total		21	43	22	16	102	102

**Table 3:** Participant profiles by professional certification.

Certification	Shiga	JCHO	Shimane	Sunagawa	Total
Registered	0	3	1	0	4
Board Certified	4	11	0	0	15
Fellow	15	11	2	0	28
Senior Fellow	6	5	3	0	14
Total	25	30	6	0	61

**Table 4:** Participant profiles by institution type.

Institution type	Shiga	JCHO	Shimane	Sunagawa	Total
Primary care	0	3	0	0	3
Hospital	10	22	1	0	33
Tertiary referral hospital	8	12	19	16	55
Other	1	1	0	0	2
NA	2	5	2	0	9
Total	21	43	22	16	102

### 3.2 Characteristics of physicians’ disease knowledge

The questionnaire utilized a 5-point scale, to facilitate the answering by physicians, as discussed. For statistical processing of the results expressed in the scale, we needed to convert the 5-point scale to a binary scale, *known* and *unknown*. This subsection outlines the procedure.

First of all, we had to verify that the 5-point scale appropriately reflected the physicians’ disease knowledge. For this purpose, we included several common diseases in the questionnaire with which the physicians would be familiar. The other diseases were those about which the physicians might or might not know. Accordingly, the answers for the common diseases should be easy to differentiate from the rest. Table 5 shows the statistics of answers for both disease classes. A chi-square test revealed that the questionnaire was able to differentiate the two classes, rejecting the null hypothesis that answer distribution for both classes were the same (*p* = 7 × 10^−17^ and *V* = 0.259, where *V* is Cramer’s *V*). This result strongly suggests that the 5-point scale reflects the physicians’ disease knowledge.

**Table 5:** Numbers of responses for the two disease categories.

	Diagnose	Explain	Seen	Imagine	Haven’t heard
Common	349	391	233	23	24
Regular	189	353	431	247	816

Next, we had to confirm whether the 5-point scale answers were being appropriately converted into binary categories. To this end, we divided the categories (*can diagnose*, *can explain*, *know the disease*, *can imagine* and *haven’t heard*) into upper and lower parts using several different thresholds, assuming that the 5-point scale was suitably ordered. Each of the division threshold was then tested to see if the statistical properties held across the possible divisions. In these tests, the threshold should exhibit significant difference between the two disease classes to demonstrate its appropriateness. Table 6 shows the results of a chi-square test with the null hypothesis that common diseases are more likely to be *unknown* than other (regular) diseases.

**Table 6:** Answer thresholds and their statistical properties.

Known vs. Unknown	p-value	Effect size (*ϕ* coefficient)
Diagnose vs Explain/Seen/Imagine/Haven’t heard	6.35 × 10^−65^	0.267
Diagnose/Explain vs Seen/Imagine/Haven’t heard	1.27 × 10^−129^	0.380
Diagnose/Explain/Seen vs Imagine/Haven’t heard	2.33 × 10^−146^	0.404
Diagnose/Explain/Seen/Imagine vs Haven’t heard	3.99 × 10^−107^	0.345

These test results suggested that one of natural splits of the 5-point disease answers into two categories, *known* and *unknown*, could be successful. The threshold that categorized *know the disease* as *known* had the highest *ϕ* coefficient among the four possible thresholds, suggesting that this was the best at differentiating common diseases from the rest. Thus, we assumed that *can diagnose*, *can explain* and *know the disease* as *known*, and *can imagine* together with *haven’t heard* as *unknown*.

In making this decision, the threshold that categorized *know the disease* as *unknown* was also a viable option, as it exhibited the second best *ϕ* coefficient. This was due to the fact that *know the disease* responses were similar in the both classes of *known* and *unknown*, suggesting the ambiguity of the word, *know the disease*, which resulted in the poor performance to differentiate the two disease categories. A physician who has examined a patient of a certain disease might give *know the disease* response, while others who have read about the disease may also select *know the disease*. The appendix further explores the relationship between the quality of physicians’ disease knowledge and the self-assessment questionnaire.

### 3.3 Differences in disease knowledge among physician groups

**Fig 4:**
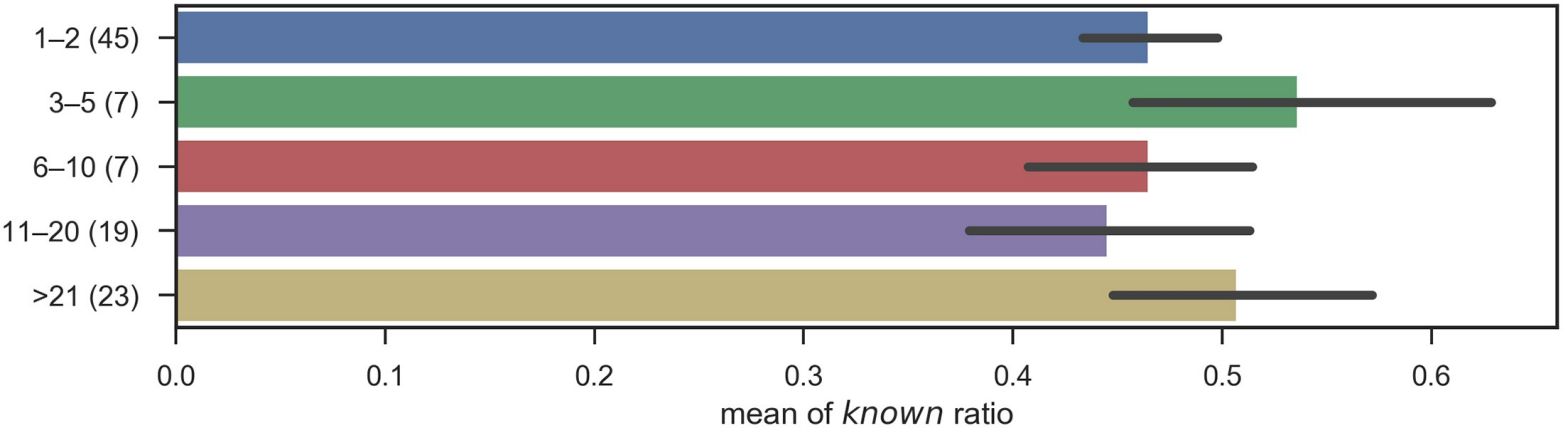
Known ratio, grouped by years of post-graduate experience: The error bars shows the standard errors.

Fig 4 shows the disease knowledge distribution when physicians were grouped by years of post-graduate experience. The number in the labels indicates the number of participants in the group. Here we have adopted the criteria for defining diseases as *known* and *unknown* given in the last section. In this subsection, raw scores are used without the correction by pseudo-diseases.

This graph suggests that there is no relationship between years of post-graduate experience and disease knowledge, as has previously been suggested [3]. To validate this statistically, a one-way variance analysis was performed to confirm that the years of post-graduate experience did not affect the responses because the result of test did not suggest statistically significant difference (*p* = 0.359 and *F*(96, 5) = 1.10).

**Fig 5:**
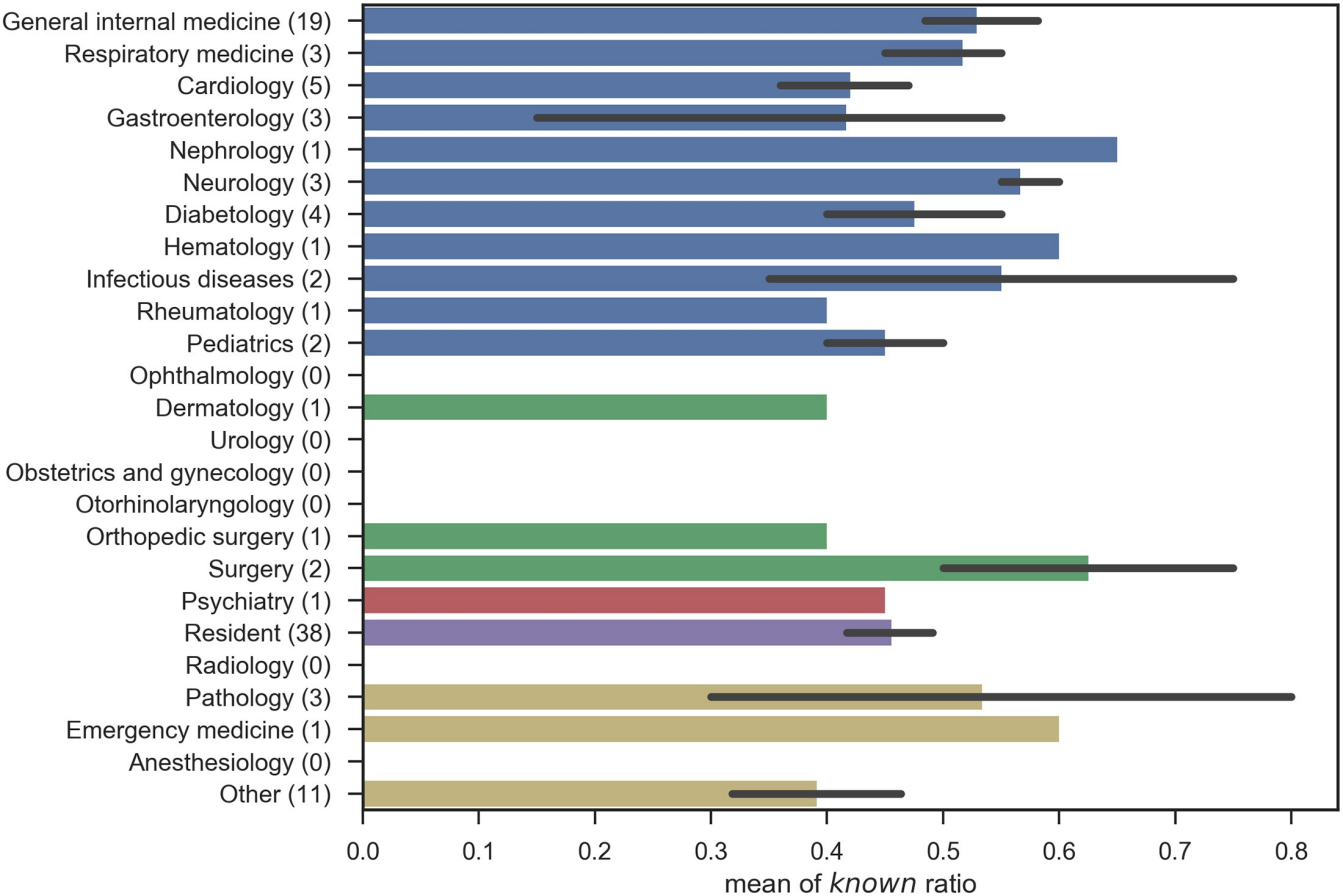
Known ratio, grouped by specialty: The error bars shows the standard errors. The bars without error bar contain only one data.

**Fig 6:**
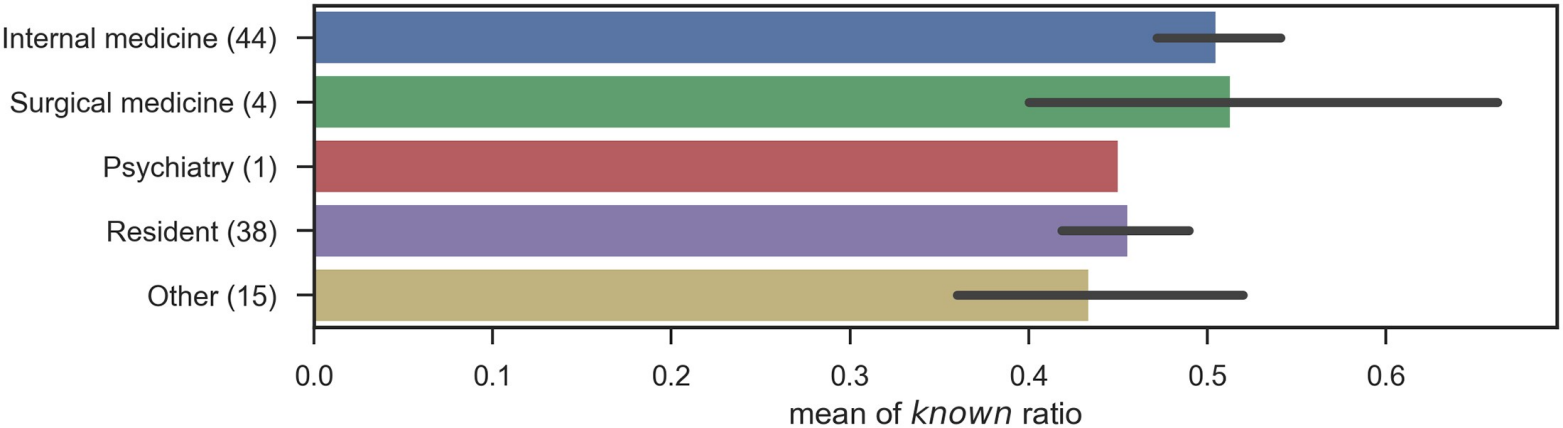
Known ratio, grouped by specialty category: The error bars shows the standard errors. The bar without error bar contains only one data.

**Fig 7:**
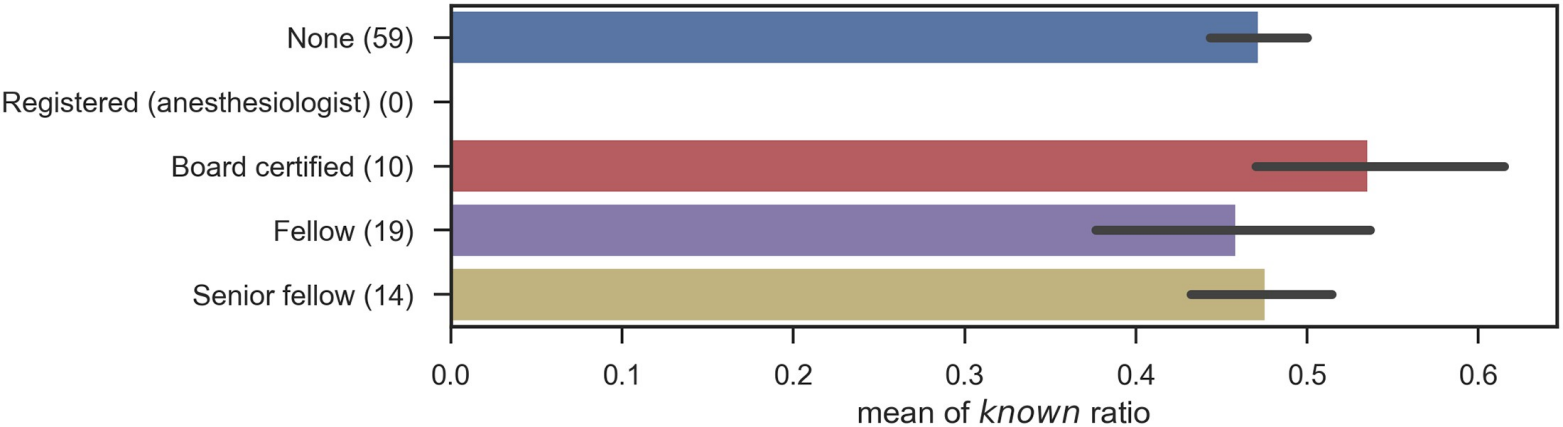
Known ratio, grouped by certification: The error bars shows the standard errors.

**Fig 8:**
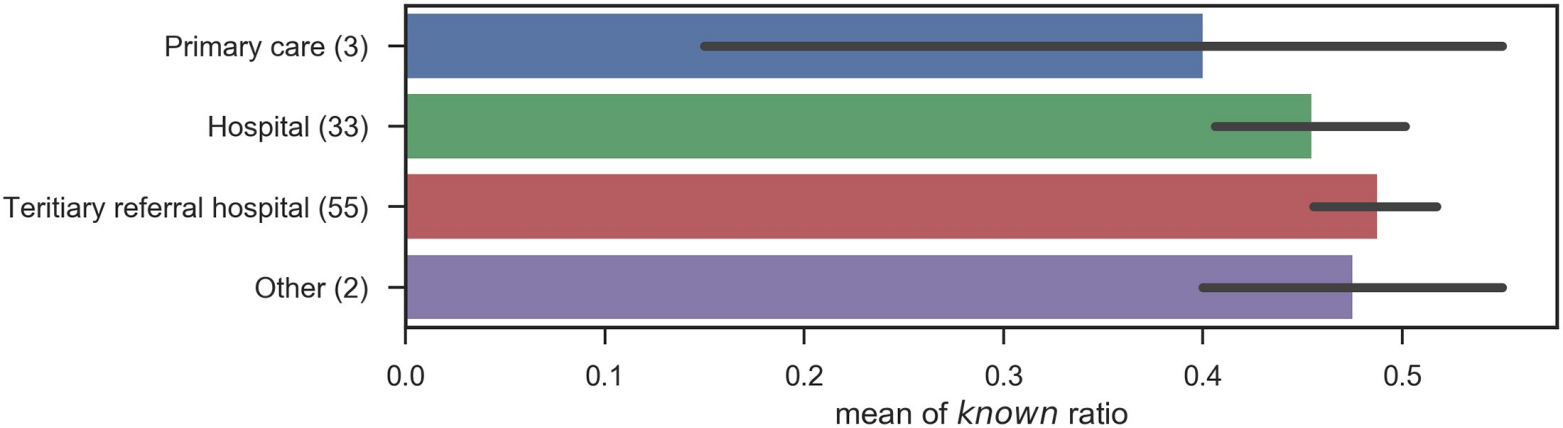
Known ratio, grouped by institution type: The error bars shows the standard errors.

Likewise, the number of *know* responses when participants were grouped by specialty, by categories of specialty, by certification, and by institution type are shown in Figs 5, 6, 7 and 8, respectively.

In these figures, no significant relation was observed between the subgroups and the number of correct answers. The results contradict with the aforementioned study [3] that proved the declines in physicians’ knowledge over time and the differences among specialties. This can mostly be ascribed to the small numbers of participants in each subgroup, as summarized in Table 7, including the degrees of freedom (inner-class and inter-class), p-values, and F-values. Note that one participant did not provide years of post-graduate experience, and the total number of participants for his group is different from that for other groups. Another possibility includes the sampling bias of test participants, because of the seminar setting we adopted. Lastly, this survey binarized physician responses, given in a 5-point scale. The scale with finer granularity might preserve the differences among groups, but, the scale is entirely subjective. This survey is designed to assess the size of vocabulary, excluding overstatement, and further assessment of the quality of physicians’ knowledge would be left for future studies.

**Table 7:** Result of one-way ANOVA for each grouping.

	Deg. of Freedom (inter-class)	Deg. of Freedom (inner-class)	F-Value	p-value
Post-graduate year	4	97	1.10	0.359
Specialty	18	82	1.21	0.267
Category of specialty	4	98	1.53	0.200
Certificate	3	97	0.870	0.459
Institution category	3	97	0.943	0.423

## 4 Analysis

### 4.1 Estimating physicians’ disease knowledge

**Fig 9:**
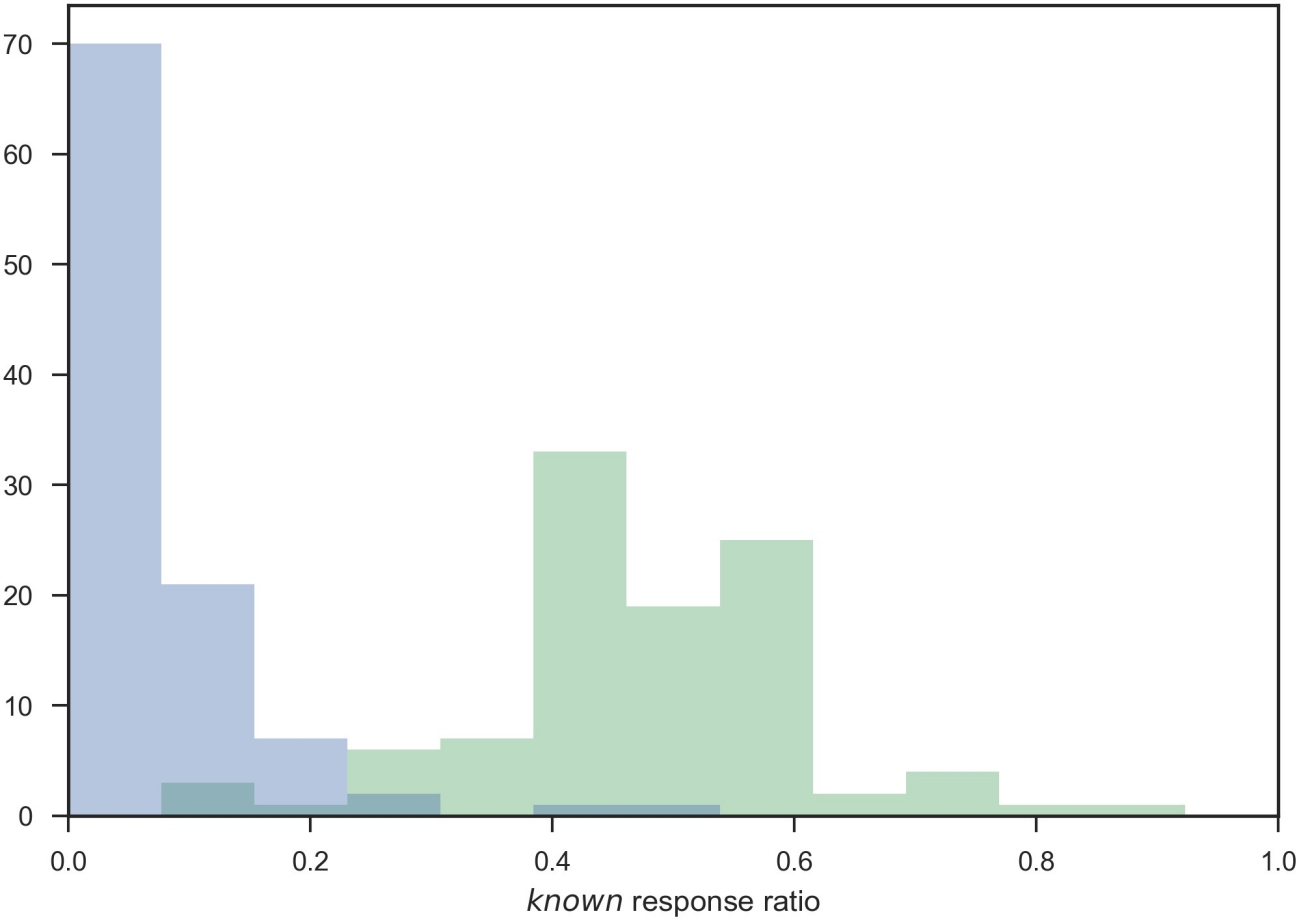
Distributions of *f*_*h*_ (*known* disease ratio: Green) and *f*_*na*_ (*known* pseudo-disease ratio: Blue) for all physicians. The dark area indicates the intersection of both.

Utilizing the results presented above, this section estimates the physicians’ level of knowledge. As discussed above, we assume that the responses *can diagnose*, *can explain*, and *know the disease* mean that the disease is *known*. The responses *haven’t heard* and *can imagine* mean that the disease is *unknown*. Now, let *f*_*h*_ be the ratio of *known* answers for diseases in the main test range. Likewise, let *f*_*na*_ be the ratio of *known* answers for pseudo-diseases. By plotting the ratios for all the test participants, we obtained histograms for *f*_*h*_ and *f*_*na*_, as shown in Fig 9. The x-axis is the ratio of *known* answers.

The probability that an average physician *knew* an arbitrary disease in the main test range was then estimated by the Clopper–Pearson method. The *known* class included 970 answers and the *unknown* class included 813, resulting in a maximum likelihood estimate for *f*_*h*_ of 0.54 (97.5% CI: (0.51, 0.54)). Likewise, there were 50 *known* answers and 856 *unknown* answers for pseudo-diseases, leading to a maximum likelihood estimate for *f*_*na*_ of 0.055 (97.5% CI: (0.039, 0.075)).

The estimated *f*_*h*_ and *f*_*na*_ values were then used to determine *f*_*k*_, the probability that the average physician knew a disease in the main test range, using Eq (1). A maximum likelihood estimate of 0.52 for *f*_*k*_ was calculated from the maximum likelihood values of *f*_*h*_ and *f*_*na*_. Similarly, 95% CI for *f*_*k*_ was (0.47, 0.56), based on the 97.5% CIs for *f*_*h*_ and *f*_*na*_.

Now, the number of diseases that the average physician knew is given, as follows. We assumed that the number of diseases all clinicians knew was 1100, and the number of diseases in the tested range was 1755. This led to an estimated average number of diseases known by physicians being 2008, with a 95% CI of (1939, 2071).

### 4.2 Coverage of the national license examination

Because this is a novel investigation in the level of disease knowledge among physicians, the estimated figure given above must be validated from various viewpoints. In this regard, the most straightforward and solid baseline is the number of diseases that appear in the license examination for physicians. Unfortunately, however, there are no known statistics for this, so instead we counted the number of diseases listed in the specification for the qualification examination for national medical practitioners in Japan. This specification is given as a huge list, hierarchically organized by topic, in PDF format [12]. This list was converted into a spreadsheet for analysis in several steps. First, some cells contained several disease names whereas others contained only one. For simplicity, all the items were extracted manually and listed on separate lines. Second, in the process, the disease names were normalized. Finally, all the items were categorized by disease name type (explained below). All processing was performed by one (T.O.) of the authors with a medical background.

The resulting list included 1902 items. However, a number of items required special considerations, which can be categorized into three types (Table 8). First of all, there are factors that underestimate the number of items counted. For example, *progressive muscular dystrophy* has several subtypes, such as Duchenne type and Becker type, which were all grouped as one disease name in the current specification. Similar cases would happen if the same disease causes different conditions in the chronic and acute phases. Another illustration is the case, “gastric ulcer/peptic ulcer”, in the specification, which could be split into separate items, gastric ulcer and peptic ulcer. This case is slightly more complex, because there is an inclusive relation between “gastric ulcer” and “peptic ulcer”, both of which are useful as disease names. Secondly, there are cases that overestimate the total number. Redundancy of items in the specification would certainly increase the total number. There are entries for a single disease, with different modifiers. Other cases included conditions triggered by certain drugs. Either case would result in overestimation. Finally, some items did not affect the total number of diseases, but were nonetheless problematic as disease names.

**Table 8:** Types of irregular entries included in the examination specification.

Type	Reason	Comments
Underestimate—*correction may increase the total*		
	Subtypes	
		From a clinical viewpoint, several disease subtypes might be differentiated.
	Supplementary names	
		Entries like “gastric ulcer/peptic ulcer” could be interpreted as two separate entries.
Overestimate—*correction may reduce the total*		
	Duplicates	
		Several diseases that appear more than once in the list, such as “polycystic ovary syndrome.”
	Diseases with modifiers	
		“Pulmonary arterial hypertension” and “pulmonary hypertension following hypoxia” might be combined. “Mucosal injury by antiplatelet agents” could also be combined with “gastritis.”
	Conditions and therapies that are not diseases	
		The list includes entries such as “emergency contraception” and “hormone replacement therapy”, which we have tentatively marked as concepts.
Others—*limited impact need to be considered*		
	Synonyms	
		“Hirschsprung’s disease” is listed together with the synonyms “congenital megacolon” and “intestinal aganglionosis.”
	Locations and conditions	
		“Tracheal stenosis,” “bronchial stenosis,” “tracheal obstruction,” and “bronchial obstruction” could be generalized as *airway obstruction*, but the etiologies might differ. Likewise, “vertebral fracture”, “pelvic fracture”, and “extremity fracture” must be managed differently, but they could otherwise be combined.
	Unqualified disease names	
		There are a few entries on the list, such as “torsion of pedicle,” that are ambiguous without further context.

To summarize, the list comprised 1902 items, including 132 duplicates and 65 disease concepts which are rough and unqualified. Although we may conclude from this that Japanese physicians need to know at least 1705 diseases, the exact number varies depending on the definition and granularity of the disease names. Nevertheless, this can be used as a minimum bound for future discussion, since we can enumerate the disease names given in the specification, and physicians are expected to know at least this number of diseases.

## 5 Discussion

### Disease familiarity distribution

The average number of diseases estimated by the protocol described in Section 2 agrees well with the expectation that typical physicians should know slightly more diseases than the number of diseases which appear in the license examination. However, Amano and Kondo [9] argued that estimation by randomly sampling a dictionary depends on chance and can only be accurate with a large sample size. Accordingly, they introduced a measure of word familiarity that assumes some deviation in the distribution to keep the questionnaire short. Read [7] also pointed out that such an approach will not accurately estimate the vocabulary size if the actual distribution is different from what is assumed. In fact, one physician in the authors (T.O.) checked through about a quarter of the whole list, and the physician did not recognize all of the diseases in the *everybody knows* range. As a result, the familiarity distribution should be investigated further, and the protocol used for determining the disease sampling range could be reconsidered.

### Fixed questionnaire items

In this survey, the questions on the questionnaire were fixed across all instances. This design runs the risk of causing errors in the estimate due to selecting diseases that are not representative in the distribution. The *known* ratio of the answers to the sampled diseases, by the above-noted author, differed to the ratio of the answers to different samples taken from the same range. We need to reduce the risk in future surveys. There are two contrasting approaches to eliminating this possibility: increasing the number of questions asked and selecting new random items for each instance. Although the randomization approach might be appropriate here, because it has the advantage of maintaining the brevity of the questionnaire, the drawback is that each item receives far fewer answers, leading to weaker statistical power for each sampled point. This is why the present study adopted the fixed question approach. In the future, however, it would be desirable to randomize the questions and to increase the number of participants, to make the estimate more robust.

### Sampling

Ramsey et al. ramsey1991 emphasized that physicians’ knowledge declines over time and suggested that procedure-oriented specialists, such as cardiologists and gastroenterologists, receive lower scores than other internists in general medical knowledge examinations. A more recent study has found that patient mortality rates for older physicians are higher than for younger physicians, even after adjusting for patient characteristics [13]. Although the sample size of our study is insufficient to validate such claims, further studies would contribute to investigate the mechanism of knowledge decay in detail. In this regard, the design of the current survey could have led to a biased selection of participants, even though the seminar approach contributed to achieving a good response rate. Future studies must aim to increase test participant diversity.

### Other limitations

This survey has focused on assessing physicians’ disease knowledge as simply as possible. We conjectured that vocabulary size could be a surrogate marker for physicians’ disease knowledge, to determine the limit. In this regard, the meaning of the term, *knowledge*, in this survey, corresponds to the lowest level, *know*, in Miller’s pyramid that classifies physicians’ clinical competence [14]. Although this assumption led to a simplified methodology for disease knowledge estimation, it might have oversimplified the idea of disease knowledge and clinical skills. It would also be necessary to evaluate the quality of physicians’ disease knowledge, as partially explored in the Appendix.

## 6 Conclusion

This paper has explored the quantitative assessment of disease knowledge among physicians. Because such a study has never been conducted before, it was necessary to design an appropriate study methodology first. Conjecturing that vocabulary assessment could be applied in this domain, we carefully designed a questionnaire and performed a preliminary survey.

For the actual survey, we prepared a questionnaire that included 20 rare diseases, 10 common diseases (as controls), and 10 pseudo-diseases. With this questionnaire, we collected 102 valid answers from 109 physicians, ranging from newly-licensed residents to veterans with more than 20 years’ experience. In the main test, the number of diseases that the average physician knew was estimated as 2008 (95% CI: (1939, 2071)). This estimate is slightly more than the number of diseases included in the specification for the national license examinations in Japan. We found no significant differences between subgroups, grouped by post-graduate years, specialty, certification level, nor institution type, in the survey we conducted.

Although there are some concerns with the final estimate, this preliminary study has made various contributions to the design of disease knowledge surveys. First, to avoid placing an unnecessary burden on physicians, we have demonstrated that self-assessment questionnaires can be used to assess disease knowledge. Second, to prevent physicians from overstating their knowledge in the self-assessment, we employed a pseudo-word approach. Third, we used paper-based tests rather than computer-based ones to further prevent participants from cheating by identifying pseudo words on the net. Fourth, we proposed to use the familiarity of diseases among physicians, to reduce the number of questions in the questionnaire. Finally, we utilized search engine statistics to approximate the familiarity, although the utility of this technique has yet to be empirically confirmed.

The assessment of physicians’ disease knowledge is one of the keys to designing clinical decision support systems, since such systems can play a complementary role by recognizing what physicians do and do not know. To the best of our knowledge, this is the first study to exploratorily addressed this problem. Although there are some rooms for improvement, it has laid the statistical foundation for the assessment of physicians’ disease knowledge. The randomized sampling of questionnaire items for each questionnaire, coupled with extended participant size, would further contribute to illuminate the process of physicians’ cognition on diseases.

## Supporting information

S1 Appendix Relationship between physicians’ disease knowledge and their self-assessment. (PDF)

S1 File Disease knowledge questionnaire (Original). (PDF)

S2 File Disease knowledge questionnaire (English). (PDF)

S3 File Data of figures and tables. (XLS)
