# Supplementary material for: Disease vocabulary size as a surrogate marker for physicians’ disease knowledge volume
Source: PLoS One. 2018 Dec 27;13(12):e0209551. doi: 10.1371/journal.pone.0209551 (PMC6307700; doi:10.1371/journal.pone.0209551)
Supplement: S1 Appendix — (PDF) [file pone.0209551.s001.pdf]

S1 Appendix

Relationship between physicians' disease knowledge and their self-assessment

S1.1 Overview

This appendix explores the relationship between physicians' disease knowledge and the result of the self-assessment questionnaire used in this article, through a supplementary survey. This survey focuses primarily on the qualitative aspects of the relationship, leaving detailed quantitative analysis for future work.

S1.2 Material and Methods

As part of the supplementary survey, test participants first take the main test, followed by an additional questionnaire regarding detailed knowledge of 20 diseases in the main test. Participants are requested to answer to all 20 items, including those they reported as "do not know." A sample sheet in the questionnaire is presented in Figure S1.

As illustrated, the questionnaire does not contain questions about signs and symptoms, which are fundamental in disease knowledge. This design might appear unreasonable; however, it is not. A disease may have various symptoms, some of which could be conditional, while others may follow earlier signs. Accordingly, a description could be complex and subsequently burden physicians participating in the study if they try to express as best as they can. Meanwhile, it is easy to classify the disease if they are aware of the signs and symptoms, and the grading also become easy if multiple-choice questions are employed. If survey participants wish to provide detailed information, they could also describe it in the pathophysiology field. Hence, we adopted the multiple-choice question.

For the diagnosis question, we may ask the diagnostic criteria. However, even a skilled physician may fail to rigorously answer a criterion, unless he specializes in the particular field. Asking questions using a diagnostic approach is more reasonable, to test whether a physician can recognize a disease or not. The same logic applies to therapeutics: the survey evaluates whether a physician can outline a therapeutic approach. The question, "other characteristics," aims to evaluate other knowledge of a disease that a physician might possess.

For grading purposes, disease classification was assigned 2 points. An individual disease may have various phenotypes, so it may fall into several classes. The questionnaire is designed to evaluate whether a physician can recognize a disease, therefore we allot 2 points if the physician marks one of the correct class. For descriptive questions, such as pathophysiology and diagnostic approach, we allot 1 point if some correct information is provided in the text and 2 points if necessary information is sufficiently covered. Four questions are listed in this category, and a perfect answer would receive 8 points. The questionnaire is marked out of 10, in total.

Disease name

Prolidase deficiency

【Classification】 ☐ Don't know

☐ Cardiovascular	☐ Respiratory	☐ Digestive system
☐ Endocrine	☐ Metabolic / Malnutrition	☐ Hematology / Immune
☐ Nervous / Cerebrovascular	☐ Infectious / Parasitic	☐ Collagen / Rheumatic
☐ Orthopedic	☐ Neoplasms / Tumor	
☐ Breast / Mammary gland	☐ Gynecological	☐ Obstetric
☐ Psychiatric	☐ Otolaryngological	☐ Urological
☐ Dermatological	☐ Ophthalmic	☐ Congenital malformations / Chromosomal

【Pathophysiology】 ☐ Don't know

【Diagnostic approach】 ☐ Don't know

【Treatment approach】 ☐ Don't know

【Other characteristics】 ☐ Don't know

Fig S1. Overview of the supplemental questionnaire

S1.3 Results

There were six volunteer physicians who attempted in the supplemental survey. Although the sample size was quite small, we confirmed that the self-assessment of disease knowledge generally correlated with the amount of disease knowledge. Additionally, we identified several factors that compromised the assessment process. In particular, the descriptive survey burdened participants, and the evaluation greatly depended on their motivation, which supports the simplified design of the self-assessment style we proposed for the main test.

Quantitative analysis

Figure S2 illustrates the relationship between the physicians' self-assessment of their disease knowledge and the extent of their disease knowledge as measured by the supplemental survey. Each physician tended to respond higher in assessments for diseases they had more knowledge about. This trend was confirmed quantitatively, as follows.

We supposed that the rank of self-assessment for a disease (from 4–*can diagnose by myself* to 0–*have not heard*) is a random variable that depends on the amount of knowledge and is analyzed using a generalized linear model (GLM). We assumed that the rank is a random variable that follows the binomial distribution. The probability that the rank of self-assessment is equal to k is

$$\frac{24}{(4-k)!k!}\{\mathrm{logit}(aq+b)\}^k\big(1-\mathrm{logit}(aq+b)\big)^{4-k} \tag{S1}$$

where q is the amount of knowledge, $\mathrm{logit}(p)$ is the logit function that $\mathrm{logit}(p) = \ln\frac{p}{1-p}$ and a and b are a coefficient of q and a intercept to estimate, respectively. The values of the coefficient and the intercept that maximize the likelihood are 0.5392 and -2.6612, respectively. The 95% CIs are $(0.439, 0.639)$ and $(-3.115, -2.208)$, respectively. Despite the small sample size, both confidence intervals, particulaly the CI of coefficient, does not contain zero value. Thus, we can conclude that the self-assessment scores of physicians reflect their disease knowledge. The line in Figure S2 signifies the parameter of the binomial distribution ($= \mathrm{logit}(aq+b)$) to the amount of knowledge (q).

Qualitative analysis

A detailed analysis of the answers revealed several factors that impacted the extent of knowledge measured by the questionnaire. First, the disease's name could be misunderstood, which may have resulted in an unintended overstatement. For example, a Japanese term, *chu-doku*, indicates both poisoning and dependence. Due to the overlap, participants described *nicotine dependence* for a question of *nicotine poisoning*, and they received a 0 score for the disorder that any physician could easily recognize.

Second, the survey clarified that physicians may sometimes fail to give detailed descriptions of major disorders. For example, *cocaine intoxication* is well known, and physicians could have easily described the symptoms. However, few physicians could sufficiently describe the pathophysiology. In these cases, physicians could have only scored the disease classification, which can contradict self-assessments of disease knowledge.

Lastly, missing marks were found in the submitted questionnaire, which could jeopardize the entire grading process. For accurate estimation of disease knowledge, the motivations of test participants matter, and it is advised to avoid conducting the surveillance before lunch break or at end of day.

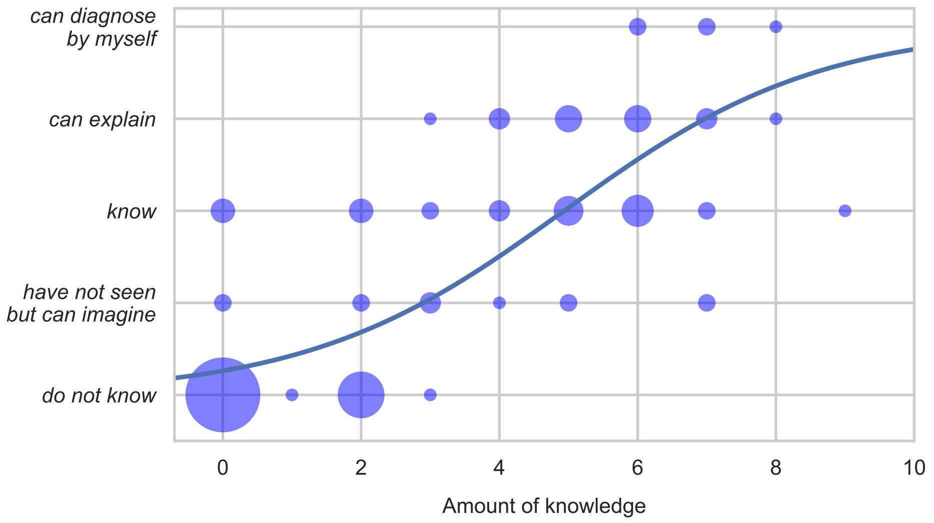

Fig S2. Distribution of the self-assessment of disease knowledge and the amount of disease knowledge. The size of each bubble is proportionate to the number of each response. The blue line indicates the mean of the point of self-assessment model under a GLM.

Participation cost

The surveillance required approximately 30 minutes, including a 10-minute main test and a 20-minute supplemental questionnaire. It is not impossible but mostly impractical, to occupy 30 minutes of physicians time for a knowledge test. The length can also discourage physicians from accurately answering the questionnaire, which could result in incorrect responses. The answer quality matters, therefore it is favorable to keep the main test as short as possible.

S1.4 Discussion

Some disease names are self-explanatory. For example, “Virus X infection” indicates that it is an infectious disease. “Substance Z deficiency” suggests that it is a Metabolic/Malnutrition disease in the questionnaire. The resulting 2 points for the classification task may appear unfair when compared to the same 2 points for a disease that is not self-explanatory, such as *Frasier syndrome*.

Such a property may distort the evaluation of physicians’ knowledge of diseases. For the appropriate correction of the issue, two factors must be considered: what diseases to correct and how to adjust the score. In this regard, disease’s names for which participating physicians selected *can imagine* are options for such correction. For the adjustment, we may also use the average score for diseases that the participants have never heard of but successfully answered a question about.

In the analysis provided below, we use “there exist some participant whose self-assessment is *do not know* but the amount of knowledge is not zero” as a criterion of self-explanatory diseases. Figure S3 illustrates the distribution of self-explanatory diseases and other diseases, and they appear to have different distributions. We adjusted the amount of self-explanatory diseases by subtracting the maximum amount of knowledge of participants whose self-assessment knowledge was “do not know” from the original amount. The lower bound was set as zero, to avoid negative knowledge.

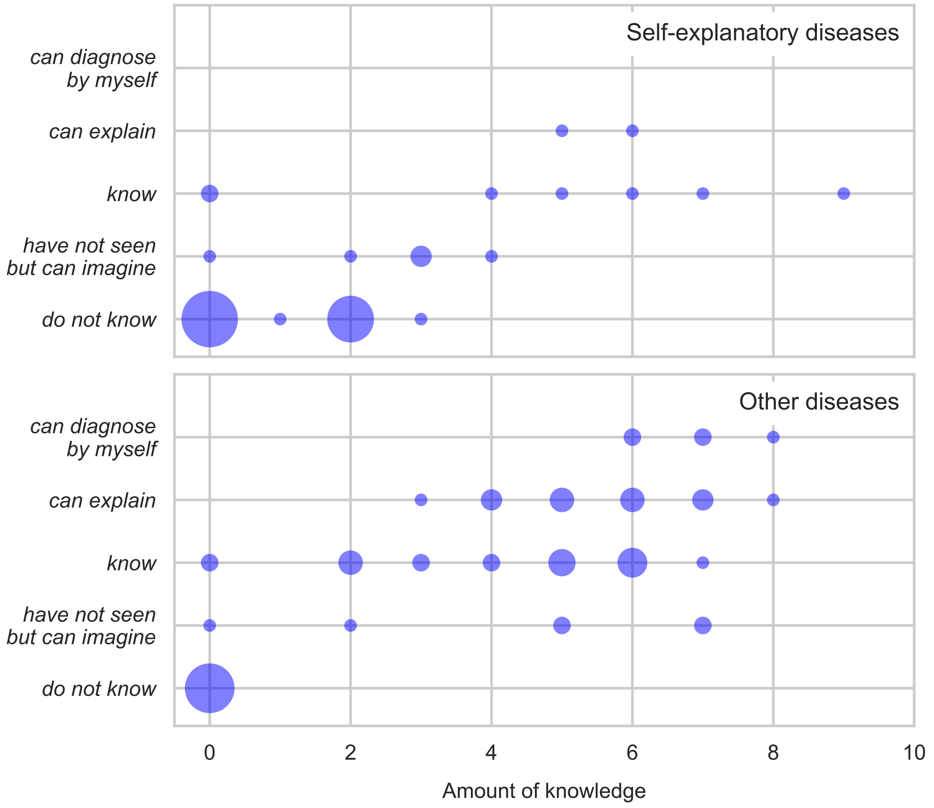

Fig S3. Respective distribution of self-assessment of knowledge and the amount of knowledge for self-explanatory diseases and other diseases. The size of each bubble is proportionate to the number of each response.

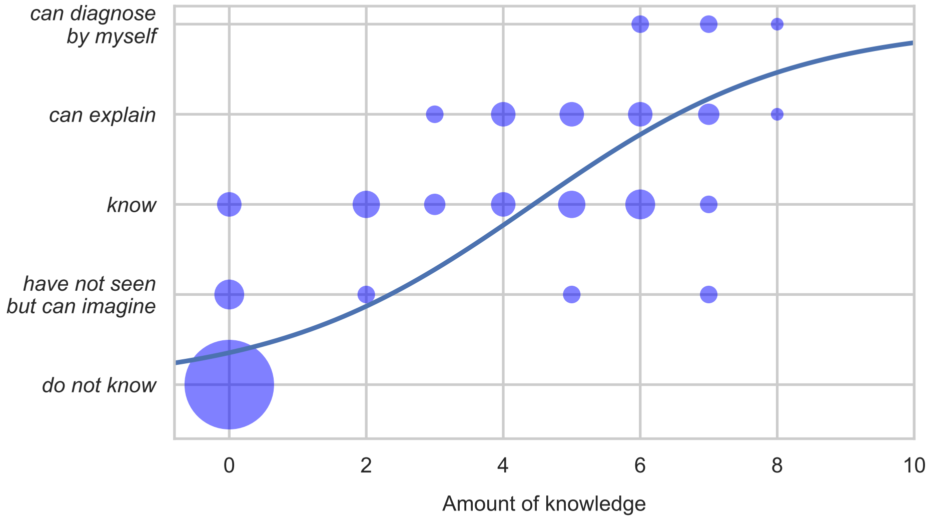

Fig S4. Distribution of assessment of disease knowledge and the amount of their corrected disease knowledge. The size of each bubble is proportionate to the number of each response. The blue line indicates the mean of the point of self-assessment model under a GLM.

Figure S4 represents the distribution of the corrected amount of knowledge and the result of self-assessment. A GLM to the corrected amount was applied again. The values of the coefficient and the intercept that brought maximum likelihood were 0.5247 and -2.3305 and their 95% CIs were $(0.432, 0.617)$ and $(-2.719, -1.943)$, respectively. We can conclude that self-assessment knowledge corresponds to the corrected amount of knowledge as presented above. Furthermore, the Bayesian information criterion (BIC) of the model for the corrected amount is -442.1, which is lower than the BIC of the model for original amount (-430.0). Resulting from this comparison, an adjustment to the amount of self-explanatory disease knowledge brings better correspondence to self-assessments of disease knowledge.

In the design process of the survey, an online questionnaire, which was easily conducted, was considered. However, it is critical that participants do not use search engines, for the validity of the study. Additionally, the survey sheet of the main questionnaire contains names for pseudo-diseases that are confidential for the survey's accuracy. Accordingly, the supplemental survey was conducted offline, coupled with the offline main test.

S1.5 Concluding Remark

This appendix attempted to clarify the relationship between physicians' disease knowledge and their responses to the self-assessment questionnaire that was designed for this article. To this end, a supplemental questionnaire was employed that measured detailed knowledge of each disease in the main test, through a multiple-choice question and in-depth description of diseases.

The question for disease classification examines basic knowledge of a disease, "what it is like". Due to the simplicity, the question did not burden physicians, and could confirm receptive (passive) vocabulary. Meanwhile, the question required special consideration for diseases with self-explanatory names, which is a drawback of the method.

The other type of question was descriptive and asked physicians to state diagnostic and therapeutic options, which are necessary for medical care. This question tested the productive (active) vocabulary of physicians. However, writing was troublesome for participating physicians and demands special consideration, such as test timing and test volume for minimizing the potential impact of impaired motivation.

The survey could not confirm the overstatement-proof property of the proposed main test, due to limited samples. In contrast, it validated that the self-assessment was well correlated with the disease knowledge of physicians. Another contribution includes the discovery of qualitative factors that influence the misunderstanding of physicians and of neglected diseases that are popular but unknown in their pathophysiology.
