# Supplementary material for: Disease vocabulary size as a surrogate marker for physicians’ disease knowledge volume
Source: PLoS One. 2018 Dec 27;13(12):e0209551. doi: 10.1371/journal.pone.0209551 (PMC6307700; doi:10.1371/journal.pone.0209551)
Supplement: S1 File — (PDF) [file pone.0209551.s002.pdf]

★マークのしかた

悪い　良い

疾患の知識量に関する調査

この調査では、40件の疾患名について、それぞれ先生がご存知か否かを自己申告して頂きます。
なお、お伺いする疾患名の中には、架空の疾患名が含まれています。
本アンケートへの回答を持って、調査へのご参加同意とさせて頂きます。ご了承下さい。

選択式の回答は、該当箇所のマークを塗りつぶしてご回答ください。この用紙は機械で処理するため、回答欄以外に書き込みをしたり、用紙を汚したり、折り目を付けたりしないようご注意ください。
: 空白マーク　: 正しいぬりつぶし　: 不十分なぬりつぶし

(1)　医師免許取得後の年数を選択して下さい
- 1〜2年目
- 3〜5年目
- 6〜10年目
- 11〜20年目
- 21年目以降

(2)　主に従事する診療科名を、１つ選択して下さい
- 内科
- 呼吸器内科
- 循環器内科
- 消化器(胃腸)内科
- 腎臓内科
- 神経内科
- 糖尿病(代謝)内科
- 血液内科
- 感染症内科
- リウマチ科
- 外科
- 整形外科
- 産婦人科
- 耳鼻咽喉科
- 麻酔科
- 小児科
- 精神科
- 泌尿器科
- 皮膚科
- 眼科
- 放射線科
- 病理診断科
- 救急科
- 臨床研修医
- その他

(3)　上記で回答した診療科について、保有している資格を選択して下さい
- 標榜医
- 認定医
- 専門医
- 指導医

(4)　主に診療に従事する医療機関の種別を選択して下さい
- 診療所 (1次)
- 病院 (2次)
- 高度医療機関 (3次)
- その他

(5)　架空のものも含む以下の各疾患に関するご自身の知識について、それぞれ最も当てはまる箇所を塗りつぶして下さい。
また、特に頻度が高い疾患や極めて稀だが医学的に重要な疾患があれば、該当欄に印をお願いします。

		疾患の知識					疾患の頻度	
		自力で診断できる	疾患を概説できる	疾患を知っている	初見だが想像できる	聞いたことがない	自身の外来で毎日診る	稀だが医学的に重要
1								
2								
3								
4								
5								
6								
7								
8								
9								
10								
11								
12								

★マークのしかた

悪い　良い

		疾患の知識					疾患の頻度	
		自力で診断できる	疾患を概説できる	疾患を知っている	初見だが想像できる	聞いたことがない	自身の外来で毎日診る	稀だが医学的に重要
13								
14								
15								
16								
17								
18								
19								
20								
21								
22								
23								
24								
25								
26								
27								
28								
29								
30								
31								
32								
33								
34								
35								
36								
37								
38								
39								
40								
