# Supplementary material for: Disease vocabulary size as a surrogate marker for physicians’ disease knowledge volume
Source: PLoS One. 2018 Dec 27;13(12):e0209551. doi: 10.1371/journal.pone.0209551 (PMC6307700; doi:10.1371/journal.pone.0209551)
Supplement: S2 File — (PDF) [file pone.0209551.s003.pdf]

Post Graduate Year ○ 1 - 2 yrs ○ 3 - 5 yrs ○ 6 - 10 yrs ○ 11 - 20 yrs ○ > 20 yrs

Speciality ________________________________

Certificate ________________________________

Affiliated institution ○ Primary ○ Secondary ○ Tertiary ○ Other

		Can diagnose	Can explain	Know the disease	Can imagine	Haven't heard	Encounter everyday	Rare but important
1	Disease 2733	○	○	○	○	○	○	○
2	Disease 7143	○	○	○	○	○	○	○
3	Disease 6057	○	○	○	○	○	○	○
4	Disease 1831	○	○	○	○	○	○	○
5	Disease 1672	○	○	○	○	○	○	○
6	Disease 6693	○	○	○	○	○	○	○
7	Disease 4464	○	○	○	○	○	○	○
8	Disease 8087	○	○	○	○	○	○	○
9	Disease 6656	○	○	○	○	○	○	○
10	Disease 7391	○	○	○	○	○	○	○
11	Disease 1334	○	○	○	○	○	○	○
12	Disease 6700	○	○	○	○	○	○	○
13	Disease 1900	○	○	○	○	○	○	○
14	Disease 7500	○	○	○	○	○	○	○
15	Disease 9101	○	○	○	○	○	○	○
16	Disease 140	○	○	○	○	○	○	○
17	Disease 630	○	○	○	○	○	○	○
18	Disease 5115	○	○	○	○	○	○	○
19	Disease 5594	○	○	○	○	○	○	○
20	Disease 3098	○	○	○	○	○	○	○
21	Disease 9648	○	○	○	○	○	○	○
22	Disease 195	○	○	○	○	○	○	○
23	Disease 3240	○	○	○	○	○	○	○
24	Disease 5197	○	○	○	○	○	○	○
25	Disease 9503	○	○	○	○	○	○	○

		Can diagnose	Can explain	Know the disease	Can imagine	Haven't heard	Encounter everyday	Rare but important
26	Disease 6047	◯	◯	◯	◯	◯	◯	◯
27	Disease 2939	◯	◯	◯	◯	◯	◯	◯
28	Disease 6097	◯	◯	◯	◯	◯	◯	◯
29	Disease 5163	◯	◯	◯	◯	◯	◯	◯
30	Disease 5640	◯	◯	◯	◯	◯	◯	◯
31	Disease 9016	◯	◯	◯	◯	◯	◯	◯
32	Disease 4320	◯	◯	◯	◯	◯	◯	◯
33	Disease 4100	◯	◯	◯	◯	◯	◯	◯
34	Disease 8018	◯	◯	◯	◯	◯	◯	◯
35	Disease 4010	◯	◯	◯	◯	◯	◯	◯
36	Disease 3574	◯	◯	◯	◯	◯	◯	◯
37	Disease 7285	◯	◯	◯	◯	◯	◯	◯
38	Disease 2533	◯	◯	◯	◯	◯	◯	◯
39	Disease 4984	◯	◯	◯	◯	◯	◯	◯
40	Disease 3917	◯	◯	◯	◯	◯	◯	◯
